# Using Canine Olfaction to Detect Bovine Respiratory Disease: A Pilot Study

**DOI:** 10.3389/fvets.2022.902151

**Published:** 2022-07-01

**Authors:** Aiden E. Juge, Nathaniel J. Hall, John T. Richeson, Courtney L. Daigle

**Affiliations:** ^1^Department of Animal Science, Texas A&M University, College Station, TX, United States; ^2^Department of Animal Science, Texas Tech University, Lubbock, TX, United States; ^3^Department of Agricultural Sciences, West Texas A&M University, Canyon, TX, United States

**Keywords:** canine, working dog, olfaction, disease detection, bovine respiratory disease, bovine, cattle

## Abstract

Bovine respiratory disease (BRD) is the leading cause of morbidity and mortality in feedlot cattle and is a major welfare and economic concern. Identification of BRD-affected cattle using clinical illness scores is problematic, and speed and cost constraints limit the feasibility of many diagnostic approaches. Dogs can rapidly identify humans and animals affected by a variety of diseases based on scent. Canines' olfactory systems can distinguish between patterns of volatile organic compounds produced by diseased and healthy tissue. In this pilot study, two dogs (“Runnels” and “Cheaps”) were trained for 7 months to discriminate between nasal swabs from cattle that developed signs of BRD within 20 days of feedlot arrival and swabs from cattle that did not develop BRD signs within 3 months at the feedlot. Nasal swabs were collected during cattle processing upon arrival to the feedlot and were stored at −80°C. Dogs were presented with sets of one positive and two negative samples and were trained using positive reinforcement to hold their noses over the positive sample. The dogs performed moderately well in the final stage of training, with accuracy for Runnels of 0.817 and Cheaps of 0.647, both greater than the 0.333 expected by chance. During a double-blind detection test, dogs evaluated 123 unique and unfamiliar samples that were presented as 41 sets (3 samples per set), with both the dog handler and data recorder blinded to the positive sample location. Each dog was tested twice on each set of samples. Detection test accuracy was slightly better than chance for Cheaps at 0.451 (95% CI: 0.344–0.559) and was no better than chance for Runnels at 0.390 (95% CI: 0.285–0.496. Overall accuracy was 0.421 (95% CI: 0.345–0.496). When dogs' consensus response on each sample set was considered, accuracy was 0.537 (95% CI: 0.384–0.689). Detection accuracy also varied by sample lot. While dogs showed some ability to discriminate between BRD-affected and healthy cattle using nasal swabs, the complexity of this task suggests that more testing is needed before determining whether dogs could be effective as a screening method for BRD.

## Introduction

Dogs' ability to detect disease *via* olfaction has been empirically evaluated for over three decades. The first publication described case reports of dogs that were alerting their owners to the presence of cancer by sniffing their leg (1), catalyzing the utilization of canines to address various contemporary health issues, including COVID-19 (2). Several dozen studies have been conducted on dogs' ability to detect a variety of diseases in humans, including cancers and bacterial infections [for a review see (3–5)]. Dogs can detect some diseases that affect cattle, including bovine viral diarrhea virus and mastitis caused by *Staphylococcus aureus* (6, 7). Many of these studies reported high sensitivity and specificity rates, suggesting that canine olfaction is a promising non-invasive screening technology for difficult-to-diagnose diseases [for a review see (8)].

One such difficult-to-diagnose condition is bovine respiratory disease (BRD). Cattle with BRD are challenging to identify and diagnose, yet cattle that remain untreated and undiagnosed can experience poor welfare. As a prey species, the evolution of cattle behavior involves the masking of behavioral indicators of disease and injury (e.g., limping, fatigue) as part of their anti-predator response (9). Furthermore, BRD involves a combination of viral and bacterial pathogens that exist commensally yet become virulent under stressful conditions (10). The prevalence of BRD in US feedlot cattle has been reported at 16.2% (11), and BRD is a leading cause of cattle morbidity and mortality worldwide due to lack of reliable testing and limited vaccine efficacy (10). The average cost of treating BRD was $23.60 per case as of 2011 (12). Given the number of animals affected, the costs of the inputs needed to treat BRD amounts to massive economic losses for the producer, notwithstanding the additional productivity and profit losses associated with morbidity, mortality, and additional labor required to administer health treatments.

Clinical illness (CI) scoring systems are the standard industry approach for identifying cattle with BRD (13). These systems use the presence or absence of specific illness indicators (e.g., nasal discharge, coughing, fever, posture, behavior) to indicate whether the animal should receive medical treatment. Several attempts at developing a standardized CI scoring system for BRD have occurred (14). The DART scoring system is a sign-based method for identifying cattle with BRD, using the indicators of behavioral depression, appetite, respiration, and temperature (15). Yet, the validity and accuracy of this approach has had limited empirical evaluation. While (16) is typically cited as the origin of DART scoring, no such system is discussed in the article itself, and DART scoring has not been evaluated in peer-reviewed literature (14). Another CI scale, known as the Wisconsin score, is discussed by (17), and uses the signs of nasal discharge, cough, eye discharge, ear position, and rectal temperature, which are scored from 0 (normal) to 3 (severe), with a total score > 4 being indicative of BRD. Love et al. (14) proposed a third CI score, referred to as the California scale, including nasal discharge, ocular discharge, rectal temperature, ear and head position, cough, and abnormal respiration, with binary classification of each category as normal or abnormal. However, both the Wisconsin score and the California scale are designed for use in young dairy calves, not adolescent beef cattle. A review of studies comparing CI scores to lung lesions in beef cattle reported that while CIS scores have an acceptable specificity of 0.92, they have low sensitivity at 0.27, suggesting that many cattle with BRD are neither diagnosed nor receive necessary health treatments (13).

Technological tools designed to identify sick cattle are in the nascent stages of development and are not yet reliable. Audio recordings of dairy calves that were used to develop an algorithm designed to automatically identify coughs had a sensitivity of 0.42 and specificity of 0.99, for cough detection with a tradeoff between sensitivity and specificity depending on the criteria for cough events (18). Automatically monitoring activity levels using an accelerometer reported that sick bulls were found to take fewer steps, have fewer bouts of lying down, lie down for less time, eat less frequently, and spend less time eating (19). When tested, the model that was developed from activity data had a sensitivity of 0.92 and specificity of 0.42, for illness detection, indicating that the tool had a greater capability for identifying sick animals but inadequate means of distinguishing healthy animals from sick ones. Our inability to reliably detect sick cattle in beef and dairy production systems using current strategies indicates that novel and innovative approaches, including the use of canine olfaction, are needed to promote cattle welfare, administer targeted antimicrobial metaphylaxis, and promote sustainability.

One promising approach involves the volatile organic compounds (VOC's) that are produced by cellular metabolism and can be detected *via* olfaction. Changes to cellular processes induced by a disease can alter the olfactory characteristics of an animal. For example, several VOCs in cattle breath are associated with BRD illness status: acetaldehyde and decanal are more frequently present in infected cattle, while methyl acetate, heptane, octanal, and several other compounds are more frequently present in healthy cattle (20). Four additional VOCs from cattle nasal mucus (i.e., phenol, benzothiazole, *p*-cresol, and 5-octadecenal), were identified as potential biomarkers of BRD (21). Two additional VOCs (i.e., 2,3-dimethyl, 1,3-pentadiene and 1,3-dimethylbutyl cyclohexane) have been identified as indicators of *Mycobacterium bovis* infection, while octadecanoic acid and hexadecenoic acid were identified as markers of uninfected cattle (22). Electronic sensor arrays consisting of multiple detectors that are each sensitive to a specific molecule have observed divergent patterns of sensor response for air from vials containing serum from healthy cattle and those inoculated with *Mannheimia haemolytica* (23). Although those sensors did not identify specific compounds associated with infection status, patterns of sensor response corresponded with peaks in acute phase proteins, including lipopolysaccharide binding protein and haptoglobin, as measured using blood tests (23). This suggests that changes in the pattern of VOCs emitted by cells are temporally linked with the immune response to pathogens and deviations in their changes may provide insight into current health status. More recently, a portable electronic VOC sensor has been developed for detection of BRD-related pneumonia in dairy calves, with perfect accuracy in an initial test (24). However, the sensor was only tested on severely ill calves. Research designed to identify olfactory molecules associated with BRD has had some success, but so far, sensors have had limited applications. Dogs might be able to provide a rapid, chute-side screening method to identify sick cattle with more capacity to detect highly stressed and immunocompromised or newly infected individuals, in addition to those already sick.

Canines have the olfactory capacity to identify patterns of VOCs and to communicate that information to humans, thus dogs may also have the capability to detect cattle affected by BRD. The goal of this pilot study was to determine whether dogs are capable of identifying cattle with BRD and cattle likely to develop BRD on the basis of nasal mucus swabs collected at feedlot entry.

## Methods

### Study Animals

Two dogs were selected from the Texas A&M University (TAMU) Comparative Pathology kennels. Runnels, a scent hound-type female, was 6 years of age at the time of the study, and arrived at the kennel facility in August 2016. Cheaps, a scent-hound type male, was 4 years of age at the time of the study, and arrived at the kennel facility in August 2018. Dogs were fed 5L18 kibble (LabDiet, St. Louis, MO, USA). Lighting was provided by fluorescent lights on a 12 h light/12 h dark schedule in addition to natural light *via* translucent panels. Dog kennels (2.5 × 1.1 × 2.5 m) were cleaned in the morning and afternoon on weekdays, and in the morning on weekends and holidays. Both dogs were previously used for clinical examination practice at the TAMU College of Veterinary Medicine and had prior experience learning basic cues *via* clicker training and positive reinforcement. A sample size of two dogs was chosen after a review of canine disease detection studies indicated that use of one or two dogs is typical in pilot studies to reduce the time investment in training (8).

### Training Room and Apparatus

**Figure 1:**
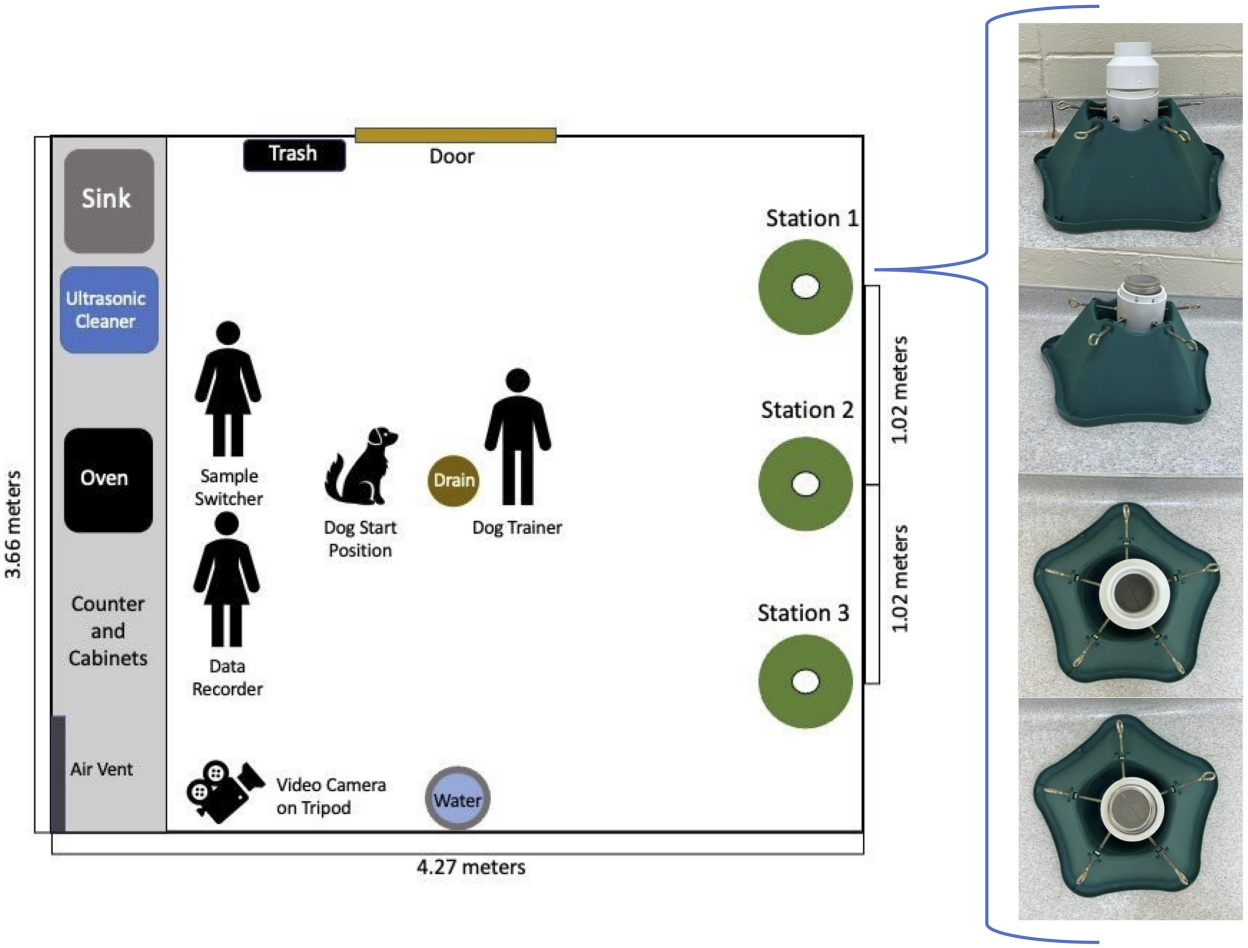
Diagram of room where training and testing were conducted. The image depicts the location and composition of the structure that was designed for this experiment.

The training room (3.66 × 4.27 m) was in a building that was separate from the kennel, had linoleum flooring, and was climate controlled to between 18 and 29°C year-round. The floor was swept and countertops cleaned daily. The training apparatus consisted of three sample stations (Figure 1). Each station consisted of a stand, with a “cup” comprised of a 7.6 cm PVC end cap and a length of 7.6 cm PVC pipe that extended out of it to form a lip (Figure 1). Detection samples were placed in 237 mL glass jars with stainless steel mesh lids, which were placed in the PVC cap, then covered with a PVC offset to prevent the dogs' noses from contacting the mesh lids. PVC offsets, jars, and lids were cleaned daily with unscented dish soap (Dawn, Proctor & Gamble, Cincinnati, OH, USA), followed by 25 min in an ultrasonic cleaner (Branson 5210, Emerson Electric Co, St Louis, MO, USA) with 50 g of low-foam powder detergent (Alcojet, Alconox, White Plains, NY, USA). After cleaning, all components were rinsed with water, and jars and lids were further decontaminated by baking in a 120°C oven (XL Convection Oven 31108, Hamilton Beach Brands, Glen Allen, VA, USA) for 15 min.

### Sample Handling

Nasal swabs were collected from 395 crossbred beef cattle at the West Texas A&M Research Feedlot in Canyon, Texas between December 2020 and March 2021. Cattle were received and processed as four lots. Samples were collected during initial processing using a cotton-tipped wooden swab. The nostril was cleaned using a paper towel, and the swab was inserted 10–15 cm into the right nostril, then rotated around the naris. Swabs were immediately placed in glass vials, clipped with scissors, and sealed with Teflon-lined plastic caps (vial # 1122-40mL, lid # 24-414, Quality Environmental Containers, Beaver, WV, USA). Samples were frozen and shipped to Texas A&M University (College Station, TX) for storage at −80°C.

After sample collection, cattle were subsequently monitored for 3 months. Samples were classified as positive if the animal's health history met two criteria: (1) the source animal was treated for BRD at least three times or died after at least one treatment for BRD, and (2) of those animals, the first treatment occurred not more than 20 days after arrival and the animal died not <20 days after arrival. These selection criteria were chosen to maximize the likelihood that cattle were in the early stages of developing BRD when the sample was collected. Samples were classified as negative if the animal was not treated for any respiratory condition and did not die within the study period. Samples from cattle that were treated once or twice, were treated more than 20 days after arrival, or died of a cause other than respiratory disease were not used. Samples from cattle that died <20 days after arrival were included as positives in testing.

Prior to initial use, sample vials were removed from the freezer (Puffer Hubbard EC8520, Revco Scientific, Asheville, NC, USA), and thawed at room temperature. Research staff wore nitrile gloves at all times when handling samples, sample jars, and PVC offsets. Clean jars and lids were wiped with methanol immediately prior to use with cattle nasal swabs. Swabs were dropped into the jars from the vial, with no direct handling, to prevent contamination with extraneous olfactory compounds. Sample jars were covered with clean metal lids when not in use. When samples were reused across multiple training sessions, the mesh jar lids were removed for cleaning and replaced with metal lids, and jars were stored in a refrigerator (Insignia NS-CF26BK6, Insignia, Richfield, MN, USA). Samples were reused for no more than five training days. Used samples were replaced in their original vials and re-frozen at −80°C for potential subsequent analysis.

### Dog Training

Three people were present during the training and testing of dogs. One person (“dog handler”) held the leash, cued the dog to search during trials and sit between trials, and provided treats for correct responses. The second person (“sample switcher”) rearranged the sample jars between trials, and the third person (“data recorder”) recorded the dog's response and indicated whether it was correct or incorrect. Dogs were trained using positive reinforcement. Dogs were presented with a lineup of three samples and were trained to perform a nose-hold alert in front of swab samples from BRD-affected cattle. Training sessions consisted of 40 trials and were held approximately five days per week. Training took place from February 2021 to August 2021, with a total of 121 training sessions per dog. All trials were recorded on a tripod-mounted video camera (Vixia HF R800, Canon, Melville, NY, USA). Sample placement for all trials was randomized using Microsoft Excel's Rand() function. Training proceeded in 10 stages (Table 1). Once a dog reached 90% accuracy within a training session, the dog moved to the next stage of training, except in stages 5–9, which were designed to expose the dogs to numerous positive and negative samples to promote generalization. However, if the dog did not perform well or exhibited a lack of participation, the dog would be returned to a previous stage of training for as long as necessary to restore performance.

**Table 1:** The stages of training used to teach the dog to detect specific samples using olfaction.

**Training stage**	**Sample set composition**	**Mean accuracy**
1	1 food vs. 2 empty jars	0.70
2	1 10^−3^ isoamyl acetate vs. 2 empty jars	0.44
3	1 10^−4^ isoamyl acetate vs. 2 empty jars	0.63
4	1 10^−5^ isoamyl acetate vs. 2 empty jars	0.89
4B	1 10^−5^ isoamyl acetate vs. 2 mineral oil	0.82
5	1 positive vs. 2 empty jars	0.98
6	1 positive vs. 2 blank swabs	0.80
7	1 positive vs. 1 negative and 1 blank swab	0.67
8	1 positive vs. 2 negative	0.63
9	1 positive vs. 2 negative, rotating	0.73
10	3 blanks, with 1 designated positive (manipulation check)	0.25
11	1 positive vs. 2 negative (familiar samples under test conditions)	0.56

Initially, dogs were presented with a lineup of three sample stations. Of these three stations, one container held food, while the other two held empty jars. Dogs were positively reinforced for using their nose to touch the station containing food. Dogs were then reinforced only for sitting after touching the target container. However, when both dogs reached Stage 4, a 1:10,000 dilution of isoamyl acetate in mineral oil, the dogs continued to make frequent mistakes. Therefore, both dogs were re-trained, beginning with Stage 1, to respond to the target scent with a nose hold of at least 4 s or at least three repeated nose touches rather than a sit alert.

Once the dogs reached 90% accuracy on Stage 4, Stage 4B was added to introduce the dogs to discriminating between similar stimuli, rather than between a stimulus and the lack of a stimulus. However, 90% accuracy in Stage 4B was not required before moving to Stage 5, since stage 4B primarily functioned to increase the difficulty of the dogs' training task while the 3-month data collection period for the first lot of cattle concluded. In Stage 5, dogs were presented with a randomly selected positive cattle nasal swab and two empty jars. This was followed by training sessions with a positive sample and two unused cotton swabs (Stage 6). In some Stage 6 training sessions, dogs were presented with two to four previously used positive samples in random order, rather than only one throughout the training session.

After meeting the criterion of 90% accuracy on several Stage 6 training sessions, the dogs progressed to Stage 7. A single randomly selected positive sample and a single randomly selected negative sample were used for 2–4 training sessions, until the dogs either reached 90% accuracy, indicating that they had learned the sample scents, or their performance decreased, potentially due to degradation of the samples. When both dogs had reached 90% accuracy on a sample set, they moved on to Stage 8, where they were presented with sets of one positive and two negative samples.

Beginning with Stage 8, trials were partially blinded. The trainer and dog faced away from the sample lineup while samples were moved between trials such that both were unaware of the location of the positive sample. The dog handler called out the dog's response, which the sample switcher confirmed as correct or incorrect. After reaching 90% accuracy on several sets of three samples, dogs moved to Stage 9. As in Stage 8, during each trial, dogs were presented with one positive and two negative samples; however, rather than presenting a single set of three samples for as long as necessary for the dogs to reach the accuracy criteria, one of the three samples was replaced every 20 trials in rotation, so that each sample was used for 60 trials. This allowed dogs to learn ten new samples each week, with the goal of promoting recognition of a BRD-specific scent. However, during stage 9, half of the trials with each sample set were unblinded, to allow the trainer to precisely reinforce clear responses and prevent the dogs from developing unwanted behaviors, such as abandoning the alert position too quickly.

On the final day of training, 10 Stage 10 trials were conducted. Trials used a set of three blank swabs to verify that dogs were not using extraneous cues to select the correct response. Additionally, 10 trials with a familiar set of one positive and two negative samples were conducted under double-blind conditions to familiarize dogs with the test procedure.

### Detection Test

Training concluded when only enough samples remained to provide adequate statistical power for testing (25). Tests were conducted in the same room as the training sessions and dogs were presented with three samples at once. However, to ensure double-blind conditions, the dog handler and dog exited the room between trials. While the dog and dog handler were outside the room, the sample switcher replaced the samples, then exited the room. The data recorder remained outside the room for the duration of the test session. After each alert, the dog handler called out which sample the dog selected, the sample switcher responded with whether the response was correct, and the dog was appropriately reinforced.

Each dog was tested on the same 32 sets of unique samples where each set consisted of one positive sample and two negative samples. Dogs were also tested on nine additional sets of samples where each set included one sample from an animal that died of respiratory illness within 20 days of feedlot arrival, and two negative samples. Sets of samples were matched for cattle shipment date to the greatest extent possible, to minimize the confound of odor differences between lots. Testing lasted for 5 days, with 20 or 22 trials per day. Dogs' starting order was balanced across test days, with one dog going first on days one and four, and the other going first on days two and three. Mesh sample jar lids were cleaned with methanol between dogs. Each dog was presented with each set of samples twice, with samples rearranged at random between trials. On the first trial with each sample set, the trial ended after the dog's first alert, which was reinforced if correct. On the second trial with each sample set, the dog was allowed as many attempts as needed to alert on the correct position, and the correct alert was reinforced. This ensured a reinforcement rate of at least 50% to minimize extinction of search behavior.

### Data Analysis

Data was reported as proportion of accurate responses and 95% confidence intervals. Accuracy was considered different than chance when chance accuracy fell outside the 95% confidence interval. Confidence intervals were calculated in Microsoft Excel Version 16.42 (Microsoft, Redmond, WA). Other results were considered significant at (*P* < 0.05). Kruskal–Wallis tests evaluated lot differences and Wilcoxon tests evaluated pair-wise comparisons and characterized sex differences in mean detection accuracy using JMP Pro 15.0 (SAS Institute Inc., Cary, NC).

Each test sample set was presented a total of four times across both dogs. To analyze dog response consistency, the number of times the dogs picked the same sample for a specific set was recorded, with possible answer outcomes including 4:0 agreement (both dogs picked the same sample during both trials), 3:1 agreement (one dog picked the same sample on one trial and the other dog picked the same sample once and a different sample once), a 2:2 split (either each dog picked the same sample twice, but selected different samples, or both dogs picked different samples on each trial, but both selected the same two samples), or a 2:1:1 split (only one sample was chosen twice across the four trials, which is the least agreement possible in a three-sample lineup). Expected values for each outcome were calculated based on the likelihood of each response split occurring by chance if both dogs chose a sample at random on each trial. A chi-square goodness of fit test was conducted using JMP Pro 15.0 to compare the distribution of response splits to expected values. To evaluate dog consensus accuracy (i.e., how accurate the dogs were when evaluated together) samples that were selected two or more times were classified as the consensus response on a sample set, with both responses in a 2:2 split weighted as 0.5 each.

## Results

### Sample Distribution

Nasal swab samples were collected from 395 cattle. Across all four cattle lots, 10 cattle died within 20 days of arrival, 70 were designated positive, 179 were designated negative, 28 were initially treated for BRD more than 20 days after arrival, 106 were treated once or twice for BRD, and 2 died of other causes. All cattle were male, with 306 bulls, 87 steers, and 2 missing sex data. Details of sample distribution and usage are presented in Table 2.

**Table 2:** Summary of the nasal swab samples that were collected from high-risk cattle upon arrival at the West Texas A&M Research Feedlot by lot, sex, health designation, and use in the training and testing phases of the study.

	**Lot 1**	**Lot 2**	**Lot 3**	**Lot 4**	**Total**
**Training phase**					96
Positive samples	23	8	4	3	38
Bull	19	6	4	3	32
Steer	4	2	0	0	6
Negative samples	20	29	5	4	58
Bull	13	17	4	4	38
Steer	7	12	1	0	20
**Testing phase**					123
Positive samples	6	0	15	11	32
Bull	6	0	12	10	28
Steer	0	0	3	1	4
Negative samples	6	15	38	23	82
Bull	5	10	25	16	56
Steer	1	5	12	7	25
Unknown	0	0	1	0	1
Died <20 Days after arrival	7	0	1	1	9
Bull	7	0	1	1	9
Not used	50	52	42	32	176
Total	112	104	105	74	395

Across samples used in training and testing, positive samples (0.857, 95% CI: 0.775–0.939) included a greater proportion of bulls than negative samples (0.671, 95% CI: 0.594–0.749). All samples from cattle that died within 20 days were from bulls.

### Training Trials

**Figure 2:**
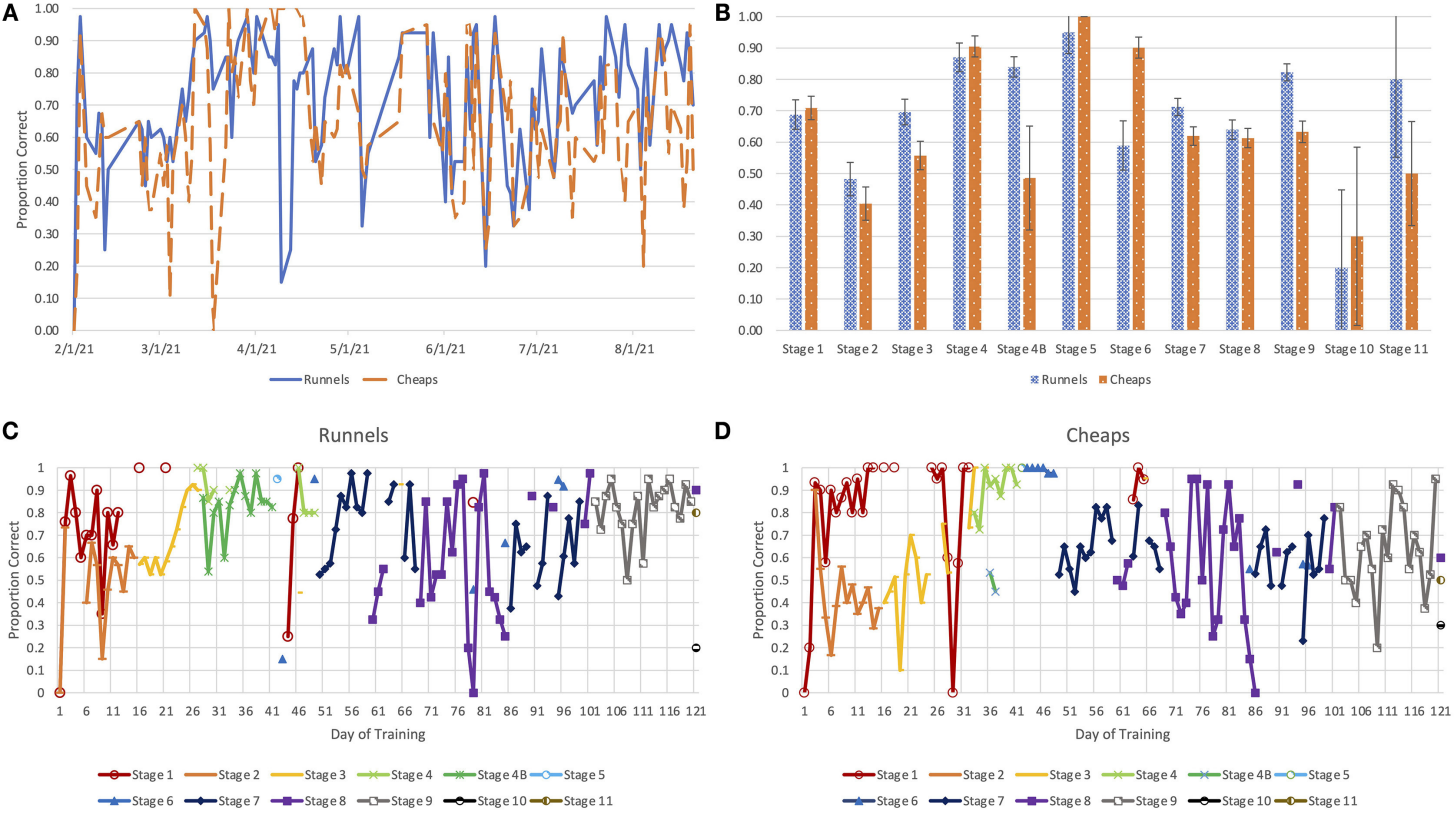
The proportion of correct responses observed **(A)** throughout the training process and **(B)** for each stage of training. Learning curves for Runnels **(C)** and Cheaps **(D)**, illustrating the proportion of correct responses for each stage of training, separated by session, although the number of trials of each stage per session varied.

Data was collected for dog performance at all stages of training (Figure 2A). Both dogs performed the best on Stage 5, where they were expected to distinguish a positive sample from empty jars. There was neither an increase in accuracy (Figure 2B) nor a consistent pattern of improvement from the first session of each training stage to the last (Figures 2C,D) for either dog. However, accuracy increased across Stages 2–4, a period that corresponded with gradually decreasing concentrations of isoamyl acetate. This suggests that the dogs readily generalized their learning of the isoamyl acetate vs. blank paradigm across concentration levels.

Accuracy levels for Stage 7 and Stage 8 fluctuated cyclically. Accuracy increased over several days of practice with each set of samples; however, accuracy decreased each time a new set of samples was introduced. The decline in accuracy on d 43 of training for Runnels and on d 30 of training for Cheaps occurred simultaneously with the training for nose hold alerts and extinction of sit alerts.

**Figure 3:**
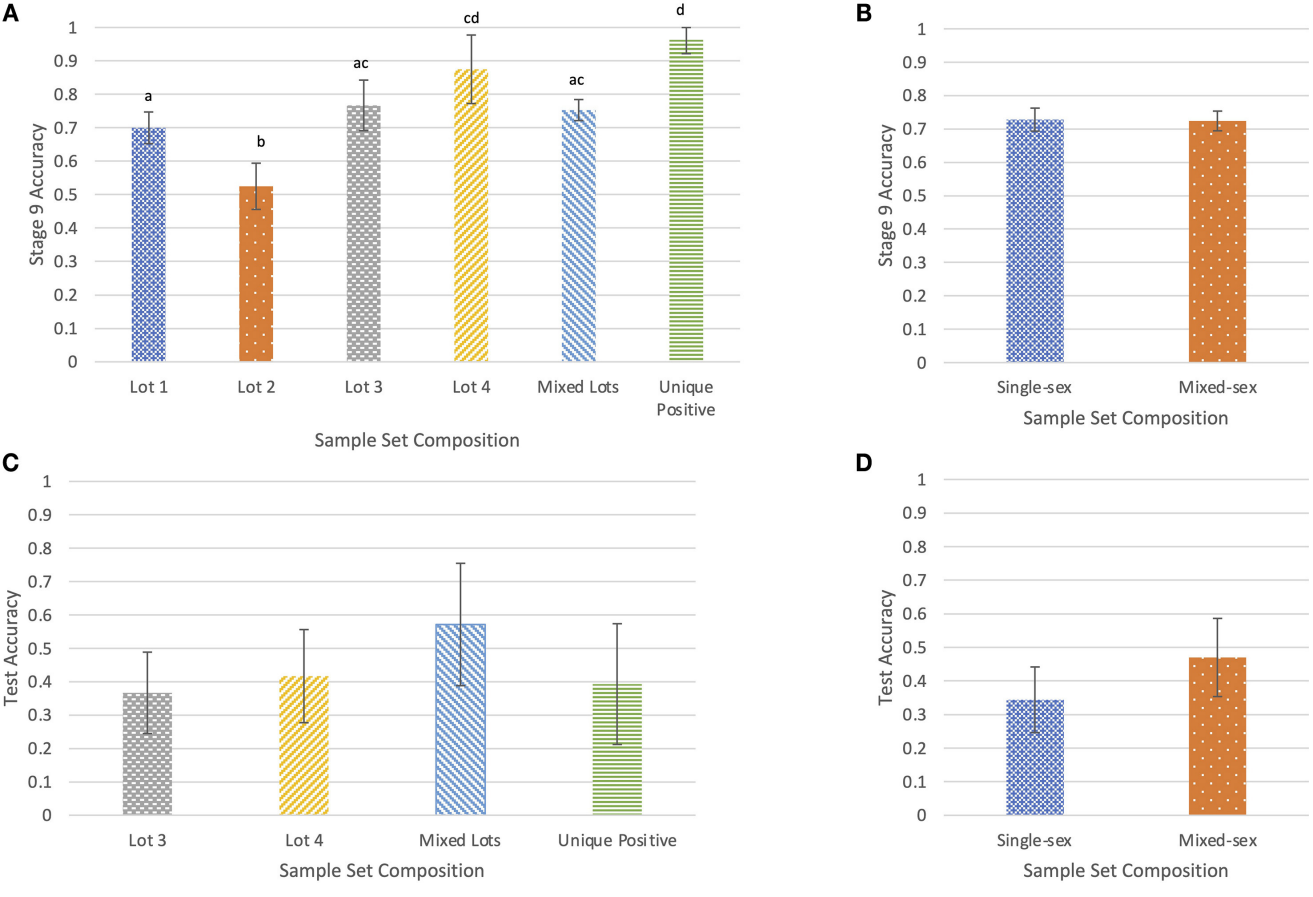
The proportion of correct responses in Stage 9 of training by lot **(A)** and by sex **(B)**, and the proportion of correct responses during the double-blind test by lot **(C)** and by sex **(D)**. Differing letters indicate significant differences at *p* < 0.05.

Mean accuracy per sample set in Stage 9 was recorded along with the lot and sex composition of the sample set. Across the 19 Stage 9 training sessions, each dog completed 20 trials with each of 38 rotating sample combinations, which included a total of 13 positive samples and 27 negative samples. Average accuracy for both dogs was 0.726 (95% CI: 0.704–0.748). Runnels had a detection accuracy of 0.818 (95% CI: 0.791–0.845), while Cheaps' detection accuracy was 0.633 (95% CI: 0.599–0.667). Mean accuracy was similar (χ^2^ = 0.090, df = 1, *p* = 0.764) for single-sex groupings of samples from all bulls, at 0.728 (95% CI: 0.694–0.762) and mixed-sex groupings of samples from both bulls and steers, at 0.724 (95% CI: 0.694–0.754) (Figure 3B).

Accuracy varied by sample lot (χ^2^ = 72.289, df = 5, *p* < 0.001; Figure 3A). For sample sets where the positive sample came from a different lot than either of the two negative samples, designated “Unique Positive,” the dogs' mean accuracy was 0.963 (95% CI: 0.922–1.00). This accuracy was greater than the mean accuracy for sets in which one negative sample came from a different lot than either of the other samples, designated “Mixed” (*p* < 0.001). The “Unique positive” accuracy was also greater than Lot 1 sample sets (*p* < 0.001), Lot 2 sample sets (*p* < 0.001), and Lot 3 samples sets (*p* < 0.001). Accuracy for Mixed sample sets was 0.753 (95% CI: 0.721–0.785) and was greater than accuracy for Lot 2 sample sets (*p* < 0.001). Within single-lot sample sets, accuracy for Lot 4 sample sets was 0.875 (95% CI: 0.773–0.977) and was greater than accuracy for both Lot 1 (*p* = 0.020) and Lot 2 sample sets (*p* < 0.001). Accuracy for Lot 3 sample sets was 0.767 (95% CI: 0.691–0.843) and was greater than accuracy for Lot 2 sample sets (*p* < 0.001). Accuracy for Lot 2 sample sets was 0.525 (95% CI: 0.456–0.594) and was lower than accuracy for Lot 1 sample sets (*p* < 0.001). Accuracy for Lot 1 sample sets was 0.700 (95% CI: 0.653–0.747).

In Stage 10, the manipulation control trials, Runnels alerted on 2/10 of the swabs arbitrarily designated as positive, and Cheaps alerted on 3/10 of them. Since this accuracy was similar to the expected chance accuracy of 0.333, it was determined that neither dog was using extraneous cues to select the correct response.

### Test Trials

Each dog completed 82 test trials. Because accuracy was similar for positive samples (0.422; 95% CI: 0.336–0.507), and samples from cattle that died within 20 days of arrival (0.417; 95% CI: 0.256–0.578), these results were combined for analysis. Detection test accuracy was low, but slightly better than the 0.333 expected by chance for Cheaps, at 0.451 (95% CI: 0.344–0.559), and was no better than chance for Runnels, at 0.390 (95% CI: 0.285–0.496). Overall accuracy was slightly better than chance at 0.421 (95% CI: 0.345–0.496). However, accuracy on the first trial with each sample was numerically lower at 0.390 (95% CI: 0.285–0.496 than on the second trial with each sample, at 0.451 (95% CI: 0.344–0.559).

Details of accuracy by sex and lot are presented in Table 3. By coincidence, both dogs had equal accuracy within each lot category for trials with single-sex sample sets. Accuracy was greater than chance for trials with mixed-sex sample sets, at 0.470 (95% CI: 0.372–0.568), but not for trials with single-sex sample sets, at 0.344 (95% CI: 0.227–0.460; Figure 3D). However, there was no difference in overall accuracy between single-sex samples sets, and mixed-sex sample sets (χ^2^ = 2.54, df = 1, *p* = 0.112). There were no differences in accuracy among sample lots (χ^2^ = 3.40, df = 3, *p* = 0.334; Figure 3C). Accuracy was numerically greatest for trials that used samples from multiple lots where at least one of the two negative samples was from the same lot as the positive sample, at 0.571 (95% CI: 0.388–0.755). Accuracy (0.367) was least for trials where all samples came from Lot 3 (95% CI: 0.245–0.489). Most samples from Lots 1 and 2 were used during training, so there were no test trials that included samples from only Lot 1 or Lot 2.

**Table 3:** Proportion correct responses on 162 total test trials using 41 sets of two negative samples and one positive sample.

**Batch difference**		**Lot 3**	**Lot 4**	**Mixed^**a**^**	**Unique positive^**b**^**	**Total**
Mixed sex		0.45	0.50	0.67	0.31	0.47
	Runnels	0.36	0.50	0.67	0.25	0.42
	Cheaps	0.55	0.50	0.67	0.38	0.52
Single sex		0.13	0.30	0.50	0.50	0.34
	Runnels	0.13	0.30	0.50	0.50	0.34
	Cheaps	0.13	0.30	0.50	0.50	0.34
Total		0.37	0.42	0.57	0.39	0.42

*Accuracy varied by sex difference in the sample set (all bulls or a mixture of bulls and steers) and by which of the four lots of cattle the samples originated from, and whether the set contained samples from more than one lot.*

*^a^Sets included one negative sample from the same lot as the positive sample.*

*^b^Sets included two negative samples from different lots than the positive sample*.

### Consistency and Consensus Accuracy

Each dog was presented with each set of samples twice, therefore, from a set of three samples, dogs would be expected to select the same response on both trials at a rate of 0.33 by chance. However, dog consistency was greater than chance, at 0.573 (95% CI: 0.466–0.680). Both dogs had similar rates of response consistency, at 0.561 (95% CI: 0.409–0.713) for Runnels and 0.585 (95% CI: 0.435–0.736) for Cheaps.

Across both dogs, each set of samples was presented four times during testing. Because there were three sample choices, the possible answer outcomes included 4:0 agreement (*n* = 2), a 3:1 split (*n* = 16), a 2:2 split (*n* = 11), or a 2:1:1 split (*n* = 12). This was compared to the distribution of response splits expected by chance if dogs chose randomly on all trials. The observed response splits did not differ from chance (χ^2^ = 3.778, df = 2, *p* = 0.286), indicating that dogs did not agree with each other at a rate greater than chance. The sample(s) selected at least twice by both dogs was classified as the dogs' consensus answer, with correct responses on sample sets with a 2:2 split weighted as 0.5. Dogs' consensus accuracy across all 41 sample sets was 0.537 (95% CI: 0.384–0.689). Consensus accuracy was similar for the 18 sample sets in which the dogs selected the same sample for three or more trials, at 0.556 (95% CI: 0.326–0.785).

### Positional Bias

During test trials, due to randomization, the proportion of times the positive sample was presented in each station varied from 0.317 to 0.366 for each dog. Runnels showed no evidence of positional bias and selected each station at a rate of 0.317–0.35. Cheaps selected station 1 at a rate of 0.195 (95% CI: 0.109–0.281), indicating a positional bias toward stations 2 and 3.

## Discussion

Both dogs performed moderately well during the training process, demonstrating that they were both able to distinguish between the scents of individual nasal swab samples and to repeatedly select a previously reinforced sample. However, this rate of correct responses was not sustained through the testing phase. One dog was able to distinguish between unfamiliar positive and negative nasal swab samples at a rate that was greater than chance. However, the other dog was not. The rate of correct response by consensus was somewhat higher than the accuracy on individual trials but was still relatively low. Dogs also appeared to employ different strategies when evaluating and selecting a sample, which may have contributed to the dogs' differing levels of detection success. Runnels selected all stations at roughly equal frequency and typically alerted on the first station sniffed, while Cheaps typically searched stations in order beginning with Station 3, leading to a disproportionately low rate of alerts on Station 1.

Dogs' ability to distinguish between sample swabs from individual cattle was supported by their above-chance rates of response consistency. The finding that each dog responded more consistently than expected by chance, but that dogs did not agree with each other more often than chance, suggests that both dogs may have learned to pick up on different scent features of the cattle samples they selected, whether BRD-related or extraneous. Alternatively, the dogs may have detected their own saliva on the sample that they selected previously. Dogs are sensitive to slight differences in sample processing, such as how samples are exposed to a test strip (26). In this study, the same samples were used for both dogs, which may have caused odor contamination across multiple sample presentations.

A pattern of inconsistent accuracy occurred throughout the training process (Figure 2). In particular, the rate of correct responses fluctuated in early food trials, possibly due to dogs' inexperience with the trial tasks early in the training period, and the potential for dogs to become fixated on the food in the sample station rather than the reinforcement for a correct alert. There is evidence that dogs may learn a detection task less rapidly and progress less consistently when food is presented alongside the target odor, compared to training in which food is presented only to reinforce correct alerts (27). However, there is a lack of literature evaluating the efficacy of a separate training stage with a food target.

In contrast to food, learning about isoamyl acetate appeared to be generalizable across concentrations, with performance during Stages 2–4 resembling an ideal asymptotic learning curve (28). Dogs' success in alerting to isoamyl acetate was unsurprising, since dogs can detect a similar chemical, amyl acetate, at concentrations as low as 1 ppb (29). The dogs' excellent performances on Stages 5–6 indicated that they quickly applied the learned detection task to selecting a positive nasal swab that was substantially different from the surrounding blank swabs, and it is possible that the initial stages of training on food and isoamyl acetate targets were not necessary. However, the inclusion of Stages 5 to 6, that were intended as a gradual increase in difficulty, may have been counterproductive. Presentation of only a positive sample may have caused an overshadowing effect. Overshadowing is the phenomenon where, when two or more novel stimuli are presented simultaneously, the most salient, or obvious stimulus is more strongly associated with the unconditioned stimulus in classical conditioning (30). Overshadowing also occurs in operant conditioning, where the more discriminable stimulus elicits increased response behavior (31). Detection dog training involves primarily operant conditioning, as the dog learns to produce a correct response to a scent stimulus to receive reinforcement. The most obvious, or salient scents to the dogs are likely to be indicators of the sample being from an animal and not blank, rather than sickness. Thus, overshadowing may have suppressed the dogs' ability to learn more subtle sickness-related scents.

Finally, once the dogs moved on to Stages 7–9, cyclic fluctuations in accuracy reflected a reliance on learning to distinguish individual samples. Sample sets were used for multiple days, and accuracy tended to increase throughout training with a single sample set. Performance decreased when new samples were introduced. The cyclical pattern of performance reflects a lack of generalized learning about a BRD-specific scent. A generalization gradient represents the decrement in responding as stimuli presented become increasingly dissimilar from stimuli an animal was trained with (32). Detection dogs must respond to samples that differ from those used in training (33). The shift from responding to a specific stimulus to responding to a category of stimuli is referred to as concept formation. To respond correctly to novel samples, dogs must identify commonalities between samples for which alerts are reinforced that distinguish them from samples for which alerts are not reinforced. In detection dogs, concept formation is particularly important when the discrimination task requires identifying a target odor amidst a mixture of odors that are similar in target and non-target samples (34). For explosives-detection dogs, training with a target scent mixed with distractor odors resulted in better generalization to novel mixtures than training with a target scent that was not mixed with distractor odors; however, performance on the mixed-odor task was initially lower, indicating that it was more difficult for the dogs than identifying a pure target scent (34). Similarly, detection of disease requires dogs to identify a disease-related odor that is mixed with background odors common to sick and healthy animals. Research in pigeons indicates that concept formation requires the use of a sufficient number of positive and negative examples in training to cause the animal to identify a common difference rather than memorizing individual examples (35). Therefore, the number of training samples used in this study may not have been sufficient to facilitate generalized learning. If dogs were able to identify a common scent associated with BRD, the decline in performance when switching to an unfamiliar set of samples would be expected to diminish and eventually disappear.

Multiple factors may have influenced the dogs' decline in performance between training and testing. Training results differed in accuracy for samples from cattle in different lots, and dogs performed best on sample sets that contained samples from multiple lots. Cattle arrived over a period of 4 months, spanning winter and early spring. Seasonal changes in weather may have contributed to olfactory differences between lots. For example, there may have been more dust in samples from cattle that arrived in drier weather. There could also be seasonal differences in VOCs due to environmental contaminants. Foliage is the largest source of natural VOC emissions and is subject to seasonal changes (36). These seasonal changes could have impacted air quality, pollen levels, and available feed ingredients. Although seasonal differences in cattle diet might also play a role, no research has quantified diet-related changes in cattle breath VOCs, and changes in the composition of a concentrate diet have been shown to have minimal effects on fecal and urine VOCs (37). The allocation of samples during training may have accounted for performance differences between lots. Samples from Lots 1 and 2 were used more frequently early in the training process, so dogs had more prior experience with them. However, samples from Lots 3 and 4 were introduced toward the end of Stage 9, on d 110 of training and d 116 of training respectively, so dogs had more experience with the training process when they encountered those sample sets.

Test data indicated that sex differences may also have played a role in the dog's ability to distinguish between samples. There are differences in pheromones produced by bulls and steers (38), and these differences may have been apparent to the dogs. The dogs may have preferentially selected samples from bulls rather than samples from sick animals. Most of the samples came from bulls, and bulls were over-represented in positive samples, thus, most of the positive samples that dogs were reinforced for alerting on were from bulls, while negative samples included more steers. This suggests that when dogs were presented with samples from both bulls and steers, the dogs may have been using sex, rather than health status, to differentiate between samples. If lot and sex scent differences were more pronounced than scent differences due to health status, these extraneous cues may have had greater salience to the dogs, and therefore became more strongly linked with reward, as predicted by the Rescorla-Wagner model of associative learning (30). As a result, the dogs may have failed to learn about odors indicative of sickness cues even if they could identify them.

Nasal swab samples may not have been an effective sample type. Nasal swabs were chosen with the rationale that nasal mucus would trap volatile organic compounds associated with respiratory illness (21); however, breath or saliva are more typically used in detection studies (8). The samples used in this study provided sufficient olfactory information to allow dogs to distinguish between individuals; however, they may not have included a sufficient concentration of BRD-associated volatile organic compounds to facilitate detection of disease. While it is possible that nasal swabs could have degraded with multiple uses, performance did not seem to change with re-use.

Accurate classification (e.g., true positive, false negative) of samples is uncertain. The health validation available for this study was treatment records. Sick animals were identified for treatment using visual sign-based screening, which typically includes factors such as lethargy and abnormal posture but may be subjective (39). BRD diagnosis from clinical signs has low sensitivity (13). Therefore, the samples may have included false negatives (e.g., sick cattle that were not identified as having BRD and thus were not treated nor classified as negative). On the day that the nasal swab sample was collected, the animal may have been sick, despite not showing signs of illness throughout the 3 month observation period. Inclusion of false-negative samples would be expected to both reduce the efficacy of training and produce apparent false-positive alerts in testing, thus reducing Runnels' accuracy.

Some samples may also have been false positives. A maximum 20-d latency between sample collection and initial BRD treatment determined whether samples were classified as positive. The 20-d interval was selected with the goal of training dogs to identify BRD before cattle developed clinical signs. Excluding animals that were treated >20 d after arrival was expected to have minimal inflammatory markers upon sample collection. However, several studies involving the use of biomarkers to predict BRD development have reported that prediction typically occurs between 1 and 9 days prior to development of clinical signs (40). As a result, some animals that were classified as BRD-positive may have had no metabolic indicators of sickness upon initial processing when the sample was collected. These false-positive samples would cause an apparent false-negative result if the dogs failed to alert.

While unlikely, it must be considered that there may be no olfactory biomarker for BRD that can be detected by dogs. Previous research has found that dogs are able to detect a variety of viral and bacterial diseases, including bovine viral diarrhea virus and mastitis caused by *S. aureus* in cattle (6, 7). However, BRD may be caused by multiple bacterial and viral pathogens (41, 42), and is partially induced by stressful events (e.g., transportation, weaning) that can affect the cattle's immune responses (43). Because of the complexity of BRD pathogenesis, if detection is reliant on a pathogen-specific odor, BRD may be more challenging than other diseases for dogs to detect. This pilot study was conducted using field conditions, which may also have influenced the overall outcome by introducing the potential confounds of lot, seasonal, and sex scent differences, thus limiting the accuracy with which BRD could be diagnosed.

Because of these uncertainties, conducting further research with increased control of sample quality, more precise timing of sample collection relative to sickness, and greater sample uniformity would be advantageous. This type of approach would require the use of a group of cattle that is homogenous in breed, sex, and origin. Further, the collection of breath or saliva samples rather than nasal swabs, combined with more rigorous diagnostic procedures to evaluate cattle health status at the time of sampling, would simplify the detection paradigm. If dogs could discriminate between samples from sick and healthy cattle under highly controlled conditions, further research could examine what compounds are responsible for those scent differences, leading to the development of sensors for BRD. The results of this research indicate that a controlled evaluation is needed. This would provide a better test of the dogs' ability to detect markers of BRD sickness in cattle as a proof of concept, which may increase the likelihood of success when encountering conditions more like those in the field.

## Data Availability Statement

The original contributions presented in the study are included in the article/Supplementary Material, further inquiries can be directed to the corresponding author/s.

## Ethics Statement

The animal study was reviewed and approved by Texas A&M University IACUC (dog training) and the West Texas A&M University IACUC (cattle sampling).

## Author Contributions

AJ contributed to study design, trained dogs, analyzed the data, and wrote the manuscript. NH contributed to study design and advised on dog training techniques. JR contributed to study design and provided cattle nasal swabs. CD contributed to study design and edited the manuscript. All authors contributed to the article and approved the submitted version.

## Funding

This work was supported by the Universities Federation for Animal Welfare Small Project Grant #26-20/21 and the Texas A&M University Triads for Transformation Round 4.

## Conflict of Interest

The authors declare that the research was conducted in the absence of any commercial or financial relationships that could be construed as a potential conflict of interest.

## Publisher's Note

All claims expressed in this article are solely those of the authors and do not necessarily represent those of their affiliated organizations, or those of the publisher, the editors and the reviewers. Any product that may be evaluated in this article, or claim that may be made by its manufacturer, is not guaranteed or endorsed by the publisher.
